# Biological Basis of Breast Cancer-Related Disparities in Precision Oncology Era

**DOI:** 10.3390/ijms25074113

**Published:** 2024-04-08

**Authors:** Anca-Narcisa Neagu, Pathea Bruno, Kaya R. Johnson, Gabriella Ballestas, Costel C. Darie

**Affiliations:** 1 Laboratory of Animal Histology, Faculty of Biology, “Alexandru Ioan Cuza” University of Iași, Carol I bvd. 20A, 700505 Iasi, Romania; 2 Biochemistry & Proteomics Laboratories, Department of Chemistry and Biochemistry, Clarkson University, Potsdam, NY 13699-5810, USA; brunop@clarkson.edu (P.B.); johnsokr@clarkson.edu (K.R.J.); ballesg@clarkson.edu (G.B.)

**Keywords:** breast cancer (BC), precision oncology, breast cancer risk (BCR), ethnicity-related disparities, age-related disparities, sex/gender-related disparities

## Abstract

Precision oncology is based on deep knowledge of the molecular profile of tumors, allowing for more accurate and personalized therapy for specific groups of patients who are different in disease susceptibility as well as treatment response. Thus, onco-breastomics is able to discover novel biomarkers that have been found to have racial and ethnic differences, among other types of disparities such as chronological or biological age-, sex/gender- or environmental-related ones. Usually, evidence suggests that breast cancer (BC) disparities are due to ethnicity, aging rate, socioeconomic position, environmental or chemical exposures, psycho-social stressors, comorbidities, Western lifestyle, poverty and rurality, or organizational and health care system factors or access. The aim of this review was to deepen the understanding of BC-related disparities, mainly from a biomedical perspective, which includes genomic-based differences, disparities in breast tumor biology and developmental biology, differences in breast tumors’ immune and metabolic landscapes, ecological factors involved in these disparities as well as microbiomics- and metagenomics-based disparities in BC. We can conclude that onco-breastomics, in principle, based on genomics, proteomics, epigenomics, hormonomics, metabolomics and exposomics data, is able to characterize the multiple biological processes and molecular pathways involved in BC disparities, clarifying the differences in incidence, mortality and treatment response for different groups of BC patients.

## 1. Introduction

In the frame of P6 medicine, which is personalized, predictive, preventive, participatory, psycho-cognitive and population-based [1], precision medicine investigates the biological basis of diseases using molecular information that emerges from different omics fields [2], allowing for a more accurate therapy of different groups of patients who are different in disease susceptibility as well as treatment response [3]. Precision oncology (PO) was defined as “the molecular profiling of tumors to identify targetable alterations” and allows for personalized treatments to improve cancer patient outcomes [4]. PO requires the discovery of predictive and prognostic biomarkers, which have been found to have racial and ethnic differences among other types of disparities, such as chronological or biological age- or sex/gender-related ones [5,6]. For example, the application of biomarkers found in serum to distinguish between BC patients and healthy people can be an important and minimally invasive tool to improve screening programs [7]. Thus, Srivastava et al. (2019) identified both race-specific serum biomarkers, such as the tyrosine kinase receptor c-Kit, retinoblastoma proteins (Rb) and vascular endothelial growth factor receptor 2 (VEGFR2), and non-race-specific serum protein biomarkers, such as pyruvate kinase 2 (Pyk2), for racial disparities in BC progression [8]. Thus, c-Kit, a receptor tyrosine kinase that induces the migration of triple-negative BC (TNBC) cells [9], has been identified as overexpressed in African American women (AAW) with BC compared to Caucasian American women (CAW) patients, while Rb, known to inhibit tumor progression, have a lower prevalence in the serum of AAW compared to CAW with BC. Moreover, c-Kit was associated with BRCA1-mutation-associated BC [8]. In addition, VEGFR2 was significantly overexpressed in AAW cancer serum compared to AAW controls, while this was not the case in CAW patients’ serum compared to CAW controls [8].

Cancer-related disparities have been mainly associated with geographical disparities [10], socioeconomic position and social inequities, also known as “social epidemiology” [11], and different racial and ethnic groups [12]. It is known that counties characterized by elevated rates of cancer mortality usually have a higher proportion of non-Hispanic-Black adults or an older population, greater poverty, and more rurality [13]. Overall, several factors that contribute to cancer health disparities are comorbidities, social stress exposure, ancestral adaptations, such as immune response at the populational level, mitochondrial function, acquired somatic mutations in oncogenes or tumor suppressor genes and dysbiosis [12].

For BC, most evidence emphasizes racial or ethnicity-related disparities. The concept of biological races, as well as racial disparities, are human inventions, being sociopolitical constructs [14], so many authors have stated that biomedical researchers and clinicians should eliminate the use of race as a biological variable [15]. However, both the public health system and scientific literature that has been written until nowadays in biomedicine fields handle the syntagma of “racial and ethnic disparities” that result from integrative interaction between patient-related intrinsic factors, such as phenotypic-, genomic-/proteomic-, metabolomic-, epiomic-, developmental- or/and evolutionary-based characteristics, and external variables, such as exposure to a natural and/or anthropized environment, psycho-socio-economic landscape or organizational and health care system factors that act on the individuals over their life course [16]. However, when discussing racial or ethnic disparities, many authors do not provide the necessary explanations for these differences [17], particularly at the level of molecular pathways and biological processes. Thus, Linnenbringer et al. (2017) integrated several multi-level hypotheses from stress biology, BC epidemiology and health disparity-related data to develop a structural perspective for emphasizing racial disparities in BC subtypes, concluding that the socially patterned psycho-social stressors, physiological and behavioral responses and genomic pathways contribute together to the increased risk of more aggressive BC and higher mortality among Black women compared to White women [18]. Many other works have shown that BC incidence and mortality rates vary across geographic regions and countries [19]. However, while the geographic region could be used to explain some genetic, biological and environmental differences [20], countries are not ideal units for the analysis of cancer rates due to variations in population size, ethnic/genetic mosaicism due to genetic mixture, socioeconomic and cultural lifestyle and many other local variable factors, so some authors recommend the use of zone design procedures in disparity-based studies [21]. Thus, evidence suggests that BC incidence is usually greater in Western countries, such as North America, Northern and Western Europe and even Australia and New Zealand, than in the majority of African and Asian nations [22]. Other authors highlighted the differences in BC incidence and mortality rates in developed countries compared to low- and middle-income countries [23].

The incidence and mortality rates of different cancers have also been associated with sex-specific disparities [24]. Overall, the incidence of different types of cancer seems to be higher in men, who are more prone to die from cancer, than in women, for relatively unknown reasons [25]. Male breast cancer (MBC) is a rare disease, so MBC and female breast cancer (FBC) are considered different entities [26], even if both sexes share some common BCR factors [27]. However, sex differences in cancer incidence have been associated with regulatory mechanisms at the genetic/molecular level and sex steroid hormones, i.e., estrogen and progesterone, which modulate gene expression in different cancers [24]. In this context, it is important to evaluate the dose–response relationships between sex steroid hormones and BCR that were most evident for postmenopausal compared to premenopausal women [28]. Dong et al. (2022) emphasized disparities in the stage at diagnosis for BC and described seven phenotypes of late-stage BC associated with a high uninsured rate, low mammography use, high area deprivation, rurality and high poverty levels [29]. Thus, in the United States, these authors showed that these phenotypes were most prevalent in southern and western states, whereas phenotypes associated with a lower percentage of late-stage diagnosis were most prevalent in the north-eastern states and select metropolitan areas [29].

Thus, the aim of this review was to deepen the understanding of BC-related disparities, mainly from a biomedical perspective.

## 2. Race- and Ethnicity-Based Disparities in Breast Cancer

**Figure 1:**
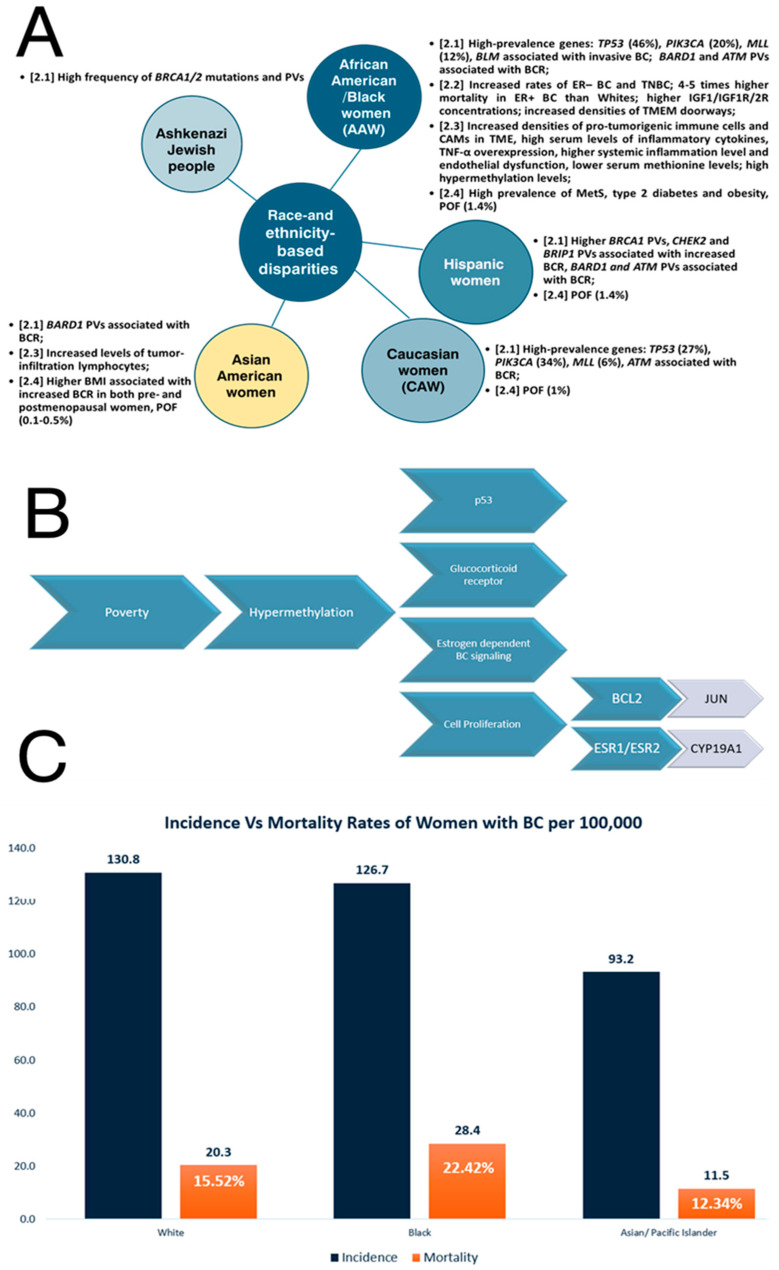
Race- and ethnicity-based disparities in breast cancer (Section 2). (**A**) disparities by race; (**B**) impact of poverty on BC incidence; (**C**) comparison of incidence versus mortality rates within women with BC. Percentage shown of mortality with reference to incidence.

Different ethnic populations are characterized by different susceptibilities to diseases [30] so group-based differences in BC incidence and mortality rates result from race and ethnicity [31]. There are many works that validate race-related differences in various organ structures and development, such as brain exposure to childhood adversity [32] or pubertal mammary gland development in conjunction with diet [33]. Generally, African American (AA)/Black individuals are known to possess a significantly greater cancer burden, with the poorest likelihood of survival leading to the worst incidence of death of any race with regard to various types of cancer [34]. Consequently, AA women (AAW)/Black women have a 41% higher mortality rate compared to White women/non-Hispanic White (NHW) women [34,35]. For BC, racial disparities are accentuated in Black women, who have a lower incidence than White and Asian women, while their BC-related mortality and aggressiveness are the highest among all races [36]. Thus, the cited incidence rates are as follows: 130.8 per 100,000 among White women, 126.7 per 100,000 among Black women, and 93.2 per 100,000 among Asian/Pacific Islander women [31]. The cited mortality rate in BC for Black women is 28 for every 100,000 individuals, White women is 20.3 for every 100,000 individuals and Asian/Pacific Islander women are 11.5 for every 100,000 individuals [31]. The incidence of BC in Native American, also known as American Indian and Alaskan Native (AI/AN), women is significantly lower than the incidence in both NHW and Black women, but the prognosis after a diagnosis of BC is worse compared to White women [37]. Several disparity-related data are summarized in Figure 1.

### 2.1. Genetics/Genomics of Breast Cancer Disparities

Overall, BC is a heterogeneous and polygenic disease [38], with 10–15% of BC cases being caused by hereditary/germline mutations in BC susceptibility genes [39], known as high-penetrance alleles/high-risk variants (i.e., *BRCA1*, *BRCA2*, *TP53*, *STK11*, *CD1* and *PTEN*), moderate-penetrance alleles/moderate-risk variants (i.e., *ATM*, *PALB2*, *CHEK2*, *BRIP1*, *RAD51C*) and common low-penetrance alleles/low-risk variants [40]. It is known that specific breast cancer 1/2 (*BRCA1/2*) mutations in the worldwide population are highly ethnic-specific [30], with a high frequency of *BRCA* variation in specific countries or ethnic groups, especially within genetically isolated populations, where these mutations are descendent from a single founder [41]. Wang (2023) summarized the main factors that contribute to the ethnic specificity of the *BRCA* variation, such as strong positive selection on human *BRCA*, adaptation to the living environment, genetic drift and founder variation in different ethnic populations [30]. *BRCA1* and *BRCA2* mutations are estimated to be responsible for about 3% of all BCs and other less common high-penetrance genes account for less than 1% of all BCs [42]. *BRCA1* and *BRCA2* genes encode proteins involved in DNA repair and homologous recombination (HR) [43], playing key roles in the maintenance of genome stability [39], including cell cycle checkpoint activations as well as transcriptional regulation and apoptosis [42]. High-penetrance germline mutations in the tumor suppressor genes result in a loss of tumor suppressor activity and an increased risk of BC [43,44]. Thus, the lifetime risk of BC in women with the *BRCA1* pathogenic mutation is 84% [44]. Of the current *BRCA* variant data, 80% were derived from European descendant populations, constituting only 20% of the world population [30]. Moreover, the mutational spectrum within *BRCA1/2* was mainly associated with an increasing risk of TNBC [38]. Thus, *BRCA1/2* pathogenic variants (PVs) have been reported in many different populations [45], like Ashkenazi Jewish people, who are at higher BCR because of a high frequency of the *BRCA1/2* gene mutations [46]. The results obtained by Bhaskaran et al. (2019) suggest that the present Caucasian population-level *BRCA* mutation signature is insufficient to accurately reflect *BRCA* status in groups other than Caucasians, for instance, people who are Chinese [39].

Somatic mutation analysis reveals racial differences in specific high-prevalence genes, such as tumor protein 53 (*TP53*) (46% in AAW vs. 27% in Caucasian women (CAW)), phosphatildylinositol-4,5-biphosphate 3-kinase (*PIK3CA*) (20% in AAW vs. 34% in CAW) and *MLL* methyltransferase family genes (12% in AAW vs. 6% in CAW) [47]. Yadav et al. (2021) performed a multigene hereditary cancer panel test for women with BC to evaluate the racial and ethnic differences in the prevalence of germline PVs and the effect of race and ethnicity on BCR among carriers [48]. These authors showed that *BRCA1* PVs were higher in Ashkenazi Jewish women and Hispanic women compared to NHW, checkpoint kinase 2 (*CHEK2*) PVs were statistically significantly lower in Black and Asian women, *BRCA1*-associated RING domain 1 (*BARD1*) PVs were associated with high BCR in Black, Hispanic, and Asian women, and ataxia-telangiectasia mutated (*ATM*) PVs were associated with increased BCR among all races and ethnicities except Asian people, whereas *CHEK2* and *BRIP1* PVs were associated with increased BCR among NHW and Hispanic women [48]. Moreover, Kwong et al. (2021) showed that the prevalence of the partner and localizer of the *BRCA2* (*PALB2*) mutation in BC also varies across different ethnic groups [49]. Germline PVs of the *PALB2* tumor suppressor gene, which binds to and co-localizes with *BRCA2* in the DNA repair pathway [50], are associated with an increased BCR, more aggressive phenotypes, particularly the TNBC subtype, and higher mortality [51]. AAW are more likely to have a basal subtype of BC and *TP53* mutations and a lower frequency of *PIK3A* mutations than White Americans [19]. DNA polymerases are also essential for DNA replication, repair mechanisms and tolerance of DNA damage [52]. Evidence suggests that DNA polymerases are associated with cancer, with many mutations in cancer cells being the result of error-prone DNA synthesis by non-replicative polymerases or the inability of replicative DNA polymerases to proofread mismatch nucleotides [53]. Family et al. (2014) analyzed single-nucleotide polymorphisms (SNPs) in DNA bypass polymerase genes, such as DNA polymerase theta (*POLQ*), and their association with BC and BC subtypes in AAW and White women, concluding that the analyzed SNPs are in high linkage disequilibrium in both races, but these can be associated with the risk of luminal BC [54]. Cells with *BRCA1/2* mutations have a homologous recombination (HRR)-deficient repair mechanism, so the poly(ADP-ribose) polymerase (PARP) inhibitors can be considered a precision-targeted anticancer drug in *BRCA1/2*-mutated women [55]. Hsiao and Lu (2021) showed that the identification of accessible homologous recombination deficiency (HRD)-type genes, which are relevant based on race, has significant clinical relevance for various malignancies, including BC [56]. Thus, these authors showed that in both White and Asian populations, more substantial mutation regions were discovered in *ATM*, *BRCA2*, the catalytic subunit of DNA polymerase epsilon (*POLE*), and type II tropoisomerase 2B (*TOP2B*), whereas variants in the replication timing regulatory factor 1 (*RIF1*), epidermal growth factor receptor (*EGFR*) and phosphatase and tensin homolog (*PTEN*) have been identified in both White and African American/Black communities. Moreover, in the African American/Black populations, there are associations of bloom syndrome helicase (*BLM*), an autosomal dominant BC susceptibility gene [57], with breast invasive carcinoma [56].

### 2.2. Breast Cancer Disparities Are Associated with Tumor Biology

The racial and ethnic differences in BC outcomes are also influenced by tumor biology [19]. Sarink et al. (2021) found that hormone receptor (HR) presence in BC prevalence varies by race/ethnicity [58]. These authors demonstrated that ER+ BCR was greater in Native Hawaiians and lower in Latina women and African Americans, although ER– BCR has increased rates in African Americans, as observed through the use of multi-ethnic cohort research [58]. Furthermore, even if the known risk variables do not entirely account for racial/ethnic variations in risk, the same authors demonstrated that relationships between obesity and oral contraceptive (OC) use with ER+ and ER− BCR differ by race/ethnicity [58]. The disparities are also particularly pronounced in ER+ BC patients, with AAW with ER+ subtype of BC experiencing four–five times higher mortality rates than their white counterparts [35].

Aberrations in insulin growth factor (IGF) signaling induced by obesity and other conditions may also contribute to racial/ethnic disparities in BC outcomes [59]. The insulin-like growth factor 1 (IGF1) axis includes insulin growth factors (IGF1 and IGF2), IGF receptors (IGF1R and IGF2R), IGF-binding proteins (IGFBPs) and IGFBP proteases [60]. IGF1 stimulates the developmental process of the mammary during fetal development, but at elevated levels, it also plays a role in the formation, progression and metastasis of BC [61,62]. It is known that IGF1 plays a key role in obesity-related endocrine cancers such as BC [63]. IGF2 is also a potent mitogen that induces cell proliferation and survival signals through activation of the IGF1 and insulin receptors (IRs), while IGF2 plasma levels are regulated by cellular uptake through IGF2R [64]. Thus, IGF1, modulated by IGF-binding protein-3 (IGFBP-3), and IGF1R were associated with stimulation of the pro-growth MAPK signal transduction pathway and the PI3K/Akt anti-apoptotic pathway that sustains BC development [62,65], so up to 50% of BC cases express the activated form of IGF1R [66]. Higgins et al. (2005) showed that numerous studies have reported higher systemic concentrations of IGF1 among AAW compared with European American women (EAW) before puberty [67]. Kalla Singh et al. (2010) showed that IGF1R’s and IGF2R’s differential expressions may contribute to an increased risk of neoplastic transformation in young AAW and to a more aggressive BC phenotype compared to CAW [64]. Moreover, Werner and Bruchim (2012) reviewed the interactions between IGF and *BRCA1* signaling pathways, emphasizing the convergence of IGF1-mediated cell survival, proliferative pathways and BRCA1-mediated tumor suppressive pathways [68].

Taking account of differences in tumor characteristics, triple-negative breast cancer (TNBC), which is the most aggressive type of BC, occurs at a higher frequency in AAW compared to CAW, even if the mutational landscape of established tumor regulatory pathway genes in TNBC seems similar [47]. Thus, 30% of BC diagnosed in AAW are TN, compared to 11–13% of non-AAW [66]. Among White women, 76% are diagnosed with the luminal A subtype of BC, while 61% of Black women have TNBC [69]. Many explanations for these disparities are based on differential familial, socioeconomic, occupational-related and medical care factors rather than on biological/biomolecular differences between races and ethnic groups [69].

Li et al. (2017) suggested that the development of personalized treatment strategies for BC patients can be improved by considering both germline and tumor-specific somatic mutations, as well as expression profiles related to drug and xenobiotic metabolizing enzymes (DXME) [70]. These authors identified significant differences among CA, AA and Asian American populations in the expression of DXME, as well as in the activation of pathways involved in commonly used chemotherapeutic drugs [70]. To exemplify, the human cytochrome P450 CYP2D6 isoform enzyme plays an important role in xenobiotic metabolism [71], and *CYP2D6* gene polymorphism can modify the pharmacokinetics of commonly used medications [72]. The frequency of *CYP2D6* alleles, which are combined at the individual level, allowing for the prediction of the metabolizer phenotype, ranging from poor metabolizer to ultra-rapid metabolizer, differs from one population to another, which explains the inter-individual differences in medication response [73].

The tumor environment (TME) has an important role in racial disparities and patient outcomes [74]. Interestingly, Kim et al. (2023) showed that, compared to White women, Black women with residual ER+ BC after neoadjuvant chemotherapy have worse distant recurrence-free survival, which can be due to a pro-metastatic TME and an increased density of “Tumor Microenvironment of Metastasis” (TMEM) doorways as portals for systemic cancer dissemination that contribute to racial disparities in BC [75]. These authors characterized the TMEM as microanatomical niches enriched for cancer stem cells (CECs) and composed of three-cell structures: a tumor cell that expresses the mammal-enabled (MENA) protein, an actin-regulatory protein involved in cell motility and adhesion [76], a tyrosine-protein kinase (TIE2)-expressing macrophage M2 and an endothelial cell, which can be together used by tumor cells as a portal to intravasate and disseminate into the bloodstream [77]. Consequently, racial differences in TMEM doorway density can contribute to racial differences in clinical outcomes [75].

### 2.3. BC Immune Landscape and BC Disparities

Evidence suggests that Black patients tend to have in their TME an increased density of pro-tumorigenic immune cells, such as M2 macrophages, which become a major population of tumor-associated macrophages (TAMs), and regulatory T cells as well as microvasculature compared to White BC patients, as a putative result of evolutionary selection for a more powerful immune response in patients with African ancestry [74]. Increased angiogenesis as well as M2 macrophages, known as tumor promotors, which support BC progression, tumor cell growth and spread, blood vessel development, cancer stem cell development, regulation of metabolic processes, and immunity resistance [78], have been correlated with increases in metastasis through the formation of TMEM. Moreover, Black patients also have high serum levels of inflammatory cytokines that sustain a pro-metastatic TME [74].

Tumor necrosis factor-α (TNF-α) is a multifunctional cytokine known as a critical regulator of inflammation and tumor progression [79]. Black women have greater TNF-α production during mid-pregnancy and lower IL-1β production postpartum [80]. It is also known that AAW tend to have higher systemic inflammation levels and endothelial dysfunction compared with CAW [81]. This can be a consequence of TNF-α overexpression, as well as other pro-inflammatory cytokines secreted by tumor and stromal cells to recruit leukocytes with metastatic effects, to generate cancer stem cells, epithelial–mesenchymal transition (EMT), invasion, resistance to therapy and metabolic reprogramming [82]. Evidence has revealed a pro-tumorigenic role of TNF-α during BC progression and metastasis [83]. Kochumon et al. (2021) showed that TNF-α activates the c-Jun NH_2_-terminal kinase (JNK/c-Jun) signaling pathway [84], promoting stem cell phenotype and tumorigenesis in TNBC through upregulation of the Notch1 signaling pathway [85], involved in normal mammary gland development as well as in BC tumorigenesis and progression [86].

Koru-Sengul et al. (2016) showed that BC in Black women exhibits a higher number of immunosuppressive cancer-associated macrophages (CAMs) with proliferative activity and a specific disposition associated with lower survival compared with non-Black Latina women and CAW [87]. Hirko et al. (2022) showed that Asian patients had increased levels of tumor-infiltration lymphocytes, reflecting disparities in the immune profile of BC in this population compared to Western patients, with applications in immune therapy [19]. Moreover, specific gene native elongation factor complex E (NELFE) with histone methyltransferase activity was associated with worse survival exclusively for AA individuals. The same authors found that methionine levels are lower in plasma samples from AAW with BC, so hypermethylation has been suggested as a possible biological/epigenetic mechanism to explain the worse outcomes in AAW with BC because many cancer suppressor genes are silenced by DNA methylation [35]. Thus, hypermethylation was correlated with high poverty levels in AAW and affects many pathways, such as p53, glucocorticoid receptor, estrogen-dependent BC signaling and cell proliferation (BCL2, JUN, ESR1, ESR2, CYP19A1) [35].

### 2.4. Metabolism-Related Disparities in Breast Cancer

Like other tumor types, BC is accompanied by metabolic reprogramming required for the proliferation, growth, invasion and migration of BC cells [88]. Attri et al. (2017) highlighted the racial disparity in the metabolic regulation of cancer [89]. Recently, Santaliz-Casiano et al. (2023) conducted a metabolomics- and bioinformatics-based study and observed that metabolic alterations are differentially associated with both AAW and NHW women, providing greater insight into the biological mechanisms underlying racial disparities in BC survival [35]. Thus, the authors observed decreased plasma levels of amino acids in AAW compared to healthy controls, while fatty acids were overexpressed in NHW patients. This study identified significant associations with regulators of metabolism, such as methionine adenosyl transferase 1A (MAT1A), DNA methyltransferase and histone methyltransferases for AAW, and fatty acid synthase (FASN) and monoacylglycerol lipase (MGL) for NHW.

Many studies have identified complex interactions between metabolic syndrome (MetS) and *BRCA1* germline mutations [61]. AAW have a high prevalence of the MetS [90] and are 70% more likely to be obese compared to NHW/CAW [91]. Also, Japanese BC patients tend to weigh more than the general population [92]. Liu et al. (2019) emphasized that, for Japanese women, a higher body mass index (BMI) was associated with an increased BCR in both pre- and postmenopausal women, while a higher BMI in Western countries was associated with an increased BCR in postmenopausal women and a decreased risk in premenopausal women [93]. Furthermore, increases in obesity, particularly abdominal obesity, and BMI are also risk factors for BC in men, in correlation to increasing estrogen levels with weight gain because of the conversion of testosterone to estrogen by aromatase in adipose tissue [94,95]. It is well known that TNBC is typically detected in young AAW and Hispanic women who carry a mutation in the *BRCA1* gene [96]. AAW have higher rates of type 2 diabetes than CAW, but paradoxically lower plasma triglycerides (TG), visceral adipose tissue and hepatic fat, and higher high-density lipoprotein (HDL) cholesterol [97]. Eketunde (2020) concluded that patients with diabetes have a higher incidence and mortality of BC due to hyperglycemia and the Warburg effect, activation of the insulin pathway, insulin-like growth factor pathways, inflammatory cytokines, and regulation of endogenous sex hormones [98]. Premature menopause, also named premature ovarian failure (POF) or premature ovarian insufficiency (POI), was reported by 1% of CAW, 1.4% of AAW, 1.4% of Hispanic women, 0.5% of Chinese women and 0.1% of Japanese women [99], with POI patients having a marginally higher insulin level [100].

## 3. Biological Sex- and Gender-Related Disparity in Breast Cancer

The incidence and mortality of different cancers have been associated with sex-specific disparities [24]. Both women and men are subject to the health effects of gender [101]. Even if these disparities happen for relatively unknown reasons, sex differences in cancer incidence have been associated with regulatory mechanisms at the genetic/molecular level and sex hormones, i.e., estrogen, which modulate gene expression in different cancers [24]. BC occurs in either gender, as female breast cancer (FBC) or male breast cancer (MBC) [102], but FBC is the principal cancer among women worldwide, whereas MBC is about 100 times less common than FBC [103]. A total of 12.9% of all women, or one in eight women, will develop BC at a certain point in their lives [31]. Consequently, BC is the most common cancer and the second highest cause of cancer death among women, while MBC is a rare disease [104], with an incidence of around 1.2 per 100,000 [105]. Thus, MBC represents less than 1% of all BC cases, accounting for 0.11% of all male neoplasms [26]. Usually, patients with MBC have it detected at an advanced stage at the time of diagnosis, are at an older age, and have a worse overall survival (OS) rate compared to FBC patients [106], so MBC mortality is higher (18.2%) than FBC (17.2%) [107]. Moreover, Zeinomar et al. (2021) showed that Black men have worse overall survival following a BC diagnosis compared to White men [108]. In the United States, rates were higher in Black men than White men for all BC subtypes, while among women, rates in Black people were 21% lower for HR+/HER2−, comparable for HR+/HER2+, 29% higher for HR−/HER2+ and 93% higher for TNBC [109].

According to mammary developmental biology, in males, breasts are rudimentary and non-functional organs, but they develop similarly in female and male fetuses [110]. In males, pubertal androgens mediate the removal of the ducts and prevention of mammary tissue development, while in females, estrogen acts as an essential regulator of branching and the development of the pubertal mammary gland [110]. This means that men have breast tissue in minimal quantity and have the potential to develop BC as well as females. FBC and MBC are considered phenotypically quite similar but different in their molecular profile [111], due to several genetic, hormonal and lifestyle/environmental risk factors [103]. In addition, a positive family history of BC is considered a major MBC predisposition factor [41]. Thus, many studies have emphasized both mutational and epigenetic similarities and differences between FBC and MBC, suggesting that some characteristics are conserved between them whereas others are not [112]. Moreover, there are hypotheses that suggest that MBC could indicate a separate form of BC that has a higher dependence on genetic variants than FBC [103]. Evidence suggests that MBC may have several distinct biological features, tending to be ductal type, luminal type A, estrogen receptor (ER)- and progesterone receptor (PR)-positive and human epidermal growth factor receptor-2 (HER2)-negative [26,113]. Moreover, increased BCR in relation to obesity has been reported both in situ and in invasive tumors, and it seems to be higher for HER2-positive than HER2-negative tumors [94,95]. Concluding here, MBC has been associated with a higher lymph node metastasis rate, higher ER positivity and lower HER2 rates [104], being considered an ER-driven BC [114]. Moreover, ERα is associated with PR in FBC, whereas Erα is associated with ERβ and the androgen receptor (AR) in MBC [113]. No luminal B or HER2 phenotypes were found in males and the basal phenotype is very rare, so male triple-negative breast cancer (TNBC) is a very rarely encountered disease [113,115].

As in FBC, high-, moderate- and low-penetrance susceptibility genes have been recognized in MBC, but these genes and their impact are not similar in FBC and MBC [41]. A total of 10% of all MBCs are hereditary forms caused by germline mutations in BC susceptibility genes [41]. Men with *BRCA1/2* mutations have an increased risk for BC: 7–8% with *BRCA2* mutations and 1% with *BRCA1* mutation, compared to 0.1% lifetime risk in the general population [116]. In addition, the *CHEK2* mutation has also been associated with an increased risk of MBC [117]. Rates of the *CHEK2* mutation seem to be higher in some countries compared to others, such as in Northern European countries, but are rare in Australia, Spain, and Ashkenazi Jewish people [117]. Szwiec et al. (2021) reviewed a lot of studies that showed that male patients with mutations in the *PALB2* gene have a seven-fold increased risk of MBC [118].

Studying the molecular differences between the FBC and MBC methylomes, Abeni et al. (2021) reported different DNA methylation levels of GTPase-related genes (RHO-GAP, RHO-GEF, and RAB GTPase) and keratin-related genes as an essential component of the cytoskeleton rearrangement biological process [119]. Known as key regulators of the cytoskeleton architecture, RHO GTPases are involved in membrane trafficking, gene transcription, cell migration, invasion, adhesion, survival and growth, and cancer initiation, metastasis and therapeutic responses [120]. Thus, Abeni et al. (2021) showed that almost all genes included in the GO term “keratin filament” were hypomethylated in FBC compared to MBC, suggesting their overexpression in FBC in association with the hypomethylation of the cytokeratin genes *KRT6A* and *KRT14* [119], which are hallmark features of TNBC [121]. These authors suggested that the overexpression of these genes has been found to be positively associated with a high tumor grade in BC and the expression of *KRT6A* and *KRT14* to be significantly associated with a basal molecular subtype of BC [119]. On the other hand, the results obtained by Callari et al. (2010) sustained a prominent role of the *AR* gene in neoplastic transformation in MBC [122]. *AR* gene maps to the X-chromosome, and X-chromosome polysomy, as well as an *AR* gene copy number increase, were emphasized in most invasive MBCs and in situ carcinomas [114]. In addition, Mule et al. (2020) demonstrated that melanoma-associated antigen A (*MAGEA*) family members, also mapped on the X-chromosome and co-regulators of *AR*, are hypomethylated in MBC, leading to their overexpression, which also suggests AR protein overexpression [114].

Chatterji et al. (2023) emphasized stanniocalcin 2 (STC2), the DEAD-box helicase family member DDX3 and the Dachshund family transcription factor 1 (DACH1) as underexploited prognostic biomarkers for MBC [111]. STC2 is a glycoprotein hormone expressed in many mammalian tissues and overexpressed in various types of cancer, including human BC, facilitating cell adaptation to stress conditions, preventing apoptosis and promoting cell proliferation, migration, immune response, tumor growth, invasion and metastasis [123]. STC2 is frequently co-expressed with ER, and it was found to be preferentially expressed in BCs of a luminal phenotype [102]. Thus, the *STC2* gene was overexpressed in MBC compared to FBC, emphasizing the greatest fold change between genders and being suggested as an independent prognostic factor for disease-free survival (DFS) in MBC [102]. Conversely, STC2 expression seems to be a favorable prognostic factor associated with extended disease-free survival and OS in FBC [102]. DDX3 is an RNA helicase with tumor suppressor and oncogenic potential, involved in cell cycle and translation regulation, DNA repair, cell survival and apoptosis [124]. The cytoplasmic DDX3 overexpression was associated with androgen expression receptor (AR), so cytoplasmic DDX3 expression could be a useful prognosticator in MBC [124]. Cui et al. (2018) showed that DACH1, which is expressed widely in normal adult tissues and functions as a tumor suppressor in a variety of neoplasms [125], is differentially expressed between MBC and FBC, concluding that the *DACH1* gene was downregulated in MBC and HER2 was overexpressed in FBC [107].

## 4. Age-Related Disparities in Breast Cancer

The elderly population is growing around the world [126] and older women are more likely to die from cancer than younger women, which in fact leads to a major health disparity [127]. It has been noted that older women have a worse prognosis compared to younger women in both early-stage and more advanced or metastatic BC [127]. Similarly, elderly MBC patients had larger tumors in more advanced stages at the time of diagnosis compared to younger patients [128]. In AAW, younger age and obesity associated with a low socioeconomic status influence TNBC development [91]. Other factors, such as age at menarche and childbearing patterns, could influence mammary gland development and BC disparity [33].

Age is one of the strongest risk factors for malignancy, due to biological changes linked to the aging process that limit health during the lifespan [129]. Consequently, outside of *BRCA* mutations, age is the main risk factor for BC development [130]. The risk of acquiring cancer-driving mutations in tissues increases as a function of chronological time [130]. Thus, genomic instability (GI), telomere shortening, epigenetic alterations, loss of proteostasis, mitochondrial dysfunction, disabled macroautophagy, deregulated nutrient sensing, cellular senescence, altered intercellular communication, chronic inflammation and dysbiosis are known as hallmarks of aging that are also hallmarks of cancer [6,131]. Moreover, Lehallier et al. (2019) showed that the patterns of changes in the proteome in different decades of life have been associated with distinct biological pathways in correlation with the genome and proteome of age-related diseases and phenotypic traits [132].

GI, known as the tendency of the genome to undergo mutations and copy number alterations (CNAs)/structural chromosome structural rearrangements/copy number variation (CNVs), is considered a hallmark of aging and is also a hallmark of BC [133,134]. DNA damage repair, DNA replication, transcription, mitotic chromosome segregation and telomere maintenance are several dysregulated biological processes that lead to GI [134]. Telomeres are essential in the maintenance of chromosome integrity and genomic stability, so telomere alteration is a feature of malignancy [135,136]. Moreover, the length of telomeres, known as repetitive sequences of DNA at the ends of chromosomes that are involved in protection against DNA degradation during cell division, is considered a biomarker of human aging and longevity [137]. Shorter relative telomere (SRT) length has been associated with both senescence and an increased BCR or with the degree of BC progression [136,138]. Interestingly, in AAW, perceived racism, a major source of chronic stress, has been inversely associated with telomere length [137]. However, Needham et al. (2020) showed that many studies found that Black people have longer leucocyte telomere lengths than White people during adulthood and suggested that race differences in telomere length may depend on socioeconomic status [139]. Furthermore, there are several studies that have emphasized that observed race differences in telomeric length are statistical artifacts [139]. However, Thorvaldsdottir et al. (2017) found that blood telomere length is predictive of BCR in *BRCA2* mutation carriers [140].

It is known that the age-associated genes in the human mammary gland drive human BC progression [141]. A study conducted by Gu et al. (2020) concluded that transcriptome changes during aging can contribute to breast tumorigenesis [141]. These authors identified 14 upregulated and 24 downregulated genes that were both age- and BC-associated. Among these deregulated genes, dynein light chain Tctex-type 3 (*DYNLT3*), prolyl 4-hydroxylase subunit alpha 3 (*P4HA3*) and Aristaless-like homeobox 4 (*ALX4*) have been identified as age-related genes that play a significant role in BC progression [141]. Thus, *DYNLT3* was found to be highly overexpressed in both BC tissues and BC cell lines, in association with N-cadherin and vimentin (VIM) overexpression associated with E-cadherin downregulation, while *DYNLT3* silencing suppressed cell growth, migration and invasion via the epithelial–mesenchymal transition (EMT) and induced cell apoptosis in MDA-MB-231 and MCF7 BC cells [142]. Interestingly, Aktary et al. (2021) showed that the level of DYNLT3 is dependent on β-catenin activity, revealing a function of the canonical Wnt/β-catenin signaling pathway during melanocyte and skin pigmentation [143]. Thus, the Wnt/β-catenin signaling pathway, which is involved in mammary development, BC cell proliferation, motility and metastasis [144], is also a central pathway in melanocyte biology [145], and it is closely associated with aging-related diseases [146]. In addition, Getz et al. (2015) suggested that the Wnt/β-catenin signaling pathway may contribute to a more aggressive phenotype present in AAW diagnosed with TNBC and could be associated with known disparities that exist in AAW compared to CAW [147]. In closing here, we may suggest an association between more aggressive BC development, black skin, and aging mediated by the Wnt/β-catenin signaling pathway, which could partially explain some biological disparities between AAW and CAW. Similarly, the *P4HA3* gene acts as an oncogene, is significantly upregulated in breast cancer, and its silencing could suppress the aggressive phenotypes of BC cells [148]. P4HA3 was also significantly upregulated in the subcutaneous adipose tissue of obese and type 2 diabetes mellitus (T2DM) patients, with a functional role in the differentiation of adipocytes and insulin resistance [149], which is known to vary across race/ethnicity [150], so AA have a high risk for T2DM and insulin resistance [151]. Similarly to DYNLT3, P4HA3 silencing significantly decreased mesenchymal markers (VIM, N-cadherin and Snail) expression and increased E-cadherin as an epithelial marker, while its overexpression produced the opposite effects, promoting cancer growth and metastasis by affecting the transforming growth factor-beta 1 (TGF-β) signaling pathway [152], which has a significant role in BC initiation and promotion and is linked to health disparities in AA [153,154]. Moreover, the TGF-β pathway enhances cell proliferation, migration, invasion and metastasis and suppresses immunosurveillance.

Black and White people’s disparities in BC mortality are most pronounced at younger ages and seem to converge later in life [31]. Thus, Hendrick et al. (2021), analyzing the age distributions of BC diagnosis and mortality by race and ethnicity in U.S. women, concluded that non-Hispanic Black, Asian American/Pacific Islander (AAPI), Native American, and Hispanic women have a higher percentage of invasive BC at younger ages and more advanced stages of BC deaths at younger ages compared to non-Hispanic White women [155]. Thus, AAW experience an increased likelihood of cancer before the age of 40, a greater severity of illnesses throughout all phases, and an elevated risk of death in comparison to White women [156]. Native American women have a younger median age of diagnosis (59 years) compared with White women (61 years) [37] and Japanese women (65 years) [92], but it is necessary to consider that Japan is the globe’s fastest aging country, where 32% of the female population were 65 or older in 2021 [157]. Moreover, many authors showed that the incidence rates in Japan have a bimodal age distribution with two peaks of 45–49 and 65–69 [157]. However, BC is the most common cancer and the second leading cause of cancer-related death in women under 40 years of age worldwide [158]. Moreover, Tzikas et al. (2020) showed that primary TNBC in younger patients is more often of a poor differentiation grade and highly proliferative compared with older patients [159]. Also, the risk of carrying a *BRCA* mutation is higher among young TNBC patients [159].

Nevertheless, in a study conducted by Metcalfe et al. (2018), the penetrance of BC in women aged 80 years, known as *BRCA* carriers, was 60.4% for those without a first-degree relative with BC and 63.3% for those with at least one first-degree relative with BC [160]. The same authors showed that the estimated penetrance of BC in women aged 80 years was 60.8% for *BRCA1* and 63.1% for *BRCA2* [160], compared with 13% of women in the general population that develop BC sometime in their lifetime. Premenopausal women emphasized a weak association between estrogen, progesterone or sex hormone-binding globulin (SHBG) levels and a positive association between androgens and breast cancer risk (BCR), while in postmenopausal women, higher estrogen and androgen levels were associated with an increase in BCR, whereas higher SHBG levels were inversely correlated with BCR [28].

## 5. Ecological Factors That Cause BC Disparities

**Figure 2:**
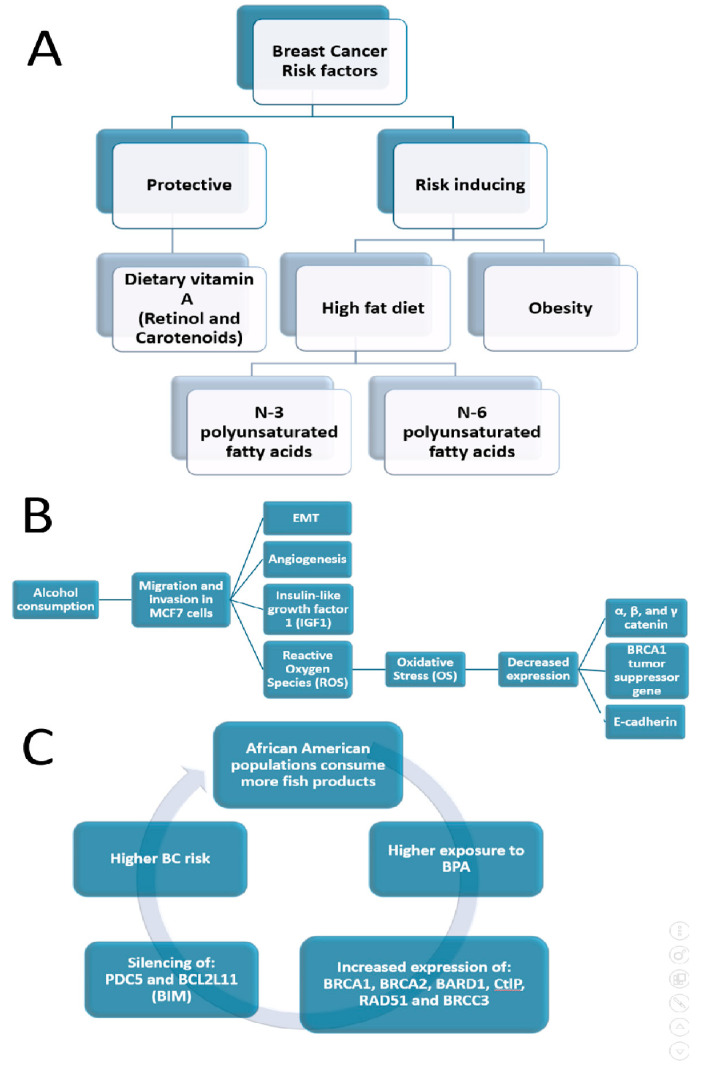
Ecological factors that cause BC disparities (Section 5). (**A**) BCR factors; (**B**) effects of alcohol consumption; (**C**) role of fish-based products on incidence of BC in AAW.

BC is commonly described as an ecological and environmental sickness or disorder [161,162,163]. To sustain this hypothesis, numerous studies have indicated that certain environmental exposures and lifestyle variables contribute 70% to 95% of the different risk factors that influence the incidence of breast cancer [164]. It is known that the higher frequency of TNBC in AAW is therefore not associated with a different genomic profile [47], as long as only 20% of TNBC tumors in AAW demonstrate *BRCA1* activity [91]. Recently, Siegel et al. (2023) showed that even cumulative exposure to neighborhood-level threat elements that particularly impact Black communities can be related to increased TNBC rates [165]. Furthermore, it was demonstrated that multiple chemicals have disproportionate exposure rates in Black women and have BC-associated biological activity as well as higher exposure-related biomarker levels than in White women [166]. Several effects of ecological factors on BC disparities are summarized in Figure 2.

### 5.1. Diet Contribution to BC-Related Disparities

It was estimated that about one-third of cancers in Western high-income societies are attributable to factors related to food, nutrition and physical activity [167]. Many food components may act as mutagens, influence the expression of oncogenes or tumor suppressor genes by the induction of epigenetic changes, such as DNA methylation or histone acetylation, and/or alter the cells’ microenvironment by modulating hormone or growth factor-based signaling, facilitating the growth and proliferation of specific cell populations [167]. Evidence suggests that a healthy dietary pattern that includes fruits and vegetables, unrefined cereals, nuts and olive oil, and a moderate or low consumption of red meat and saturated fatty acids might improve the overall survival (OS) of BC [168]. Krisanits et al. (2020) studied pubertal mammary gland development in NHW, AAW and Asian American women in combination with diets linked to changes in BC risk and disparity [33]. These authors discovered an increased BCR and BC discrepancy related to regimens comprised of high fat, N-3 polyunsaturated fatty acids, N-6 polyunsaturated fatty acids, being overweight and a Western style of eating, which can lead to abnormalities in the growth of mammary glands during puberty in mouse models [33]. Jacobs et al. (2021) indicated that both conventional meals and cereal–dairy breakfast eating patterns may minimize BCR for this population [169]. Findings on Black women as well as in women of European descent showed an inverse association of dietary vitamin A (retinol and carotenoids) intake with BCR, mainly in premenopausal women [170].

### 5.2. Alcohol Intake and BC Disparity

Higher alcohol consumption has been associated with an increased risk for BC development and appears to have a stronger effect on ER+ BC [171], because alcohol induces alterations in estrogen receptor (ER) physiology and function [172]. Women, as well as rodents used in experiments, demonstrated an elevation in estrogen (17-β-estradiol (E2)) associated with increased alcohol drinking [173], followed by the activation of ER-alpha (ER-α) [174]. Moreover, in BC cells, ERα is involved in the genomic pathway, when it is localized in the nucleus, or in a non-genomic pathway, when it is present in the cytoplasm, but in both cases, it binds to E2 [174]. The nuclear pathways of ERα action involve interaction with AP-1/c-Jun, NF-κB, p53, SP-1 and STAT [174]. Among these pathways, neuropeptide substance P (SP) and its related receptor neurokinin-1 receptor (NK1R) are known to promote the proliferation of BC cells via NF-κB-mediated inflammatory responses [175]. In addition, ERα36, an isoform of ERα that can be considered an oncogenic biomarker, is distributed in the cytoplasm and can induce the proliferation and endocrine resistance of BC cells [174]. Candelaria et al. (2015), based on a genomic-based approach, demonstrated that alcohol promotes cell proliferation and increased growth factor signaling through a large number of alcohol-responsive genes, principally those involved in apoptotic and cell proliferation pathways [176]. These authors identified the proto-oncogene *BRAF*, an alcohol- and estrogen-induced gene, to be overexpressed in BC patients with poor outcomes [176]. Moreover, Voordeckers et al. (2020) emphasize the mechanisms underlying ethanol-related genome stability by the recruitment of error-prone DNA polymerases, known as mutagenic effectors of DNA repair pathways [177], to the replication fork [178].

Heavy drinking among younger Black women was lower than that of White and Hispanic women [172], which could be associated with a lower BC incidence among Black women compared to White women [179]. Alcohol stimulates the migration and invasion of the BC cell line MCF7 [171], the EMT, vascular development, cellular oxidative stress (OS) and rendering of reactive oxygen species [180,181], with decreased levels of E-cadherin, α, β and γ catenin protein and the *BRCA1* gene, which suppresses tumor expression [171]. Furthermore, drinking alcohol affects numerous genes related to the reaction to hormonal treatment and reduces the activity of tamoxifen in BC cells [176]. Moreover, alcohol abuse interferes with insulin-like growth factor-1 (IGF1), a known contributor to pubertal development, so the alcohol delays the time of puberty in both sexes [182]. It is known that breast development and hormonal changes at puberty might affect BCR [183].

### 5.3. Endocrine Disruptor Chemicals (EDCs) and BC Disparities

James-Todd et al. (2016) showed that non-White people have higher concentrations of many EDCs compared to White people due to the higher levels of exposure and magnification that occur across their lifespans and could lead to disparate health outcomes [20]. To expand on this, non-Hispanic Black and Mexican American women have higher metabolite concentrations of low-molecular-weight phthalates (e.g., dibutyl phthalate (DBP)) than non-Hispanic White women [20]. It is known that certain phthalates that resemble estradiol, a treatment for menopausal symptoms, may induce breast cancer; for example, DBP exposure was associated with an approximately two-fold increase in the rate of ER+ BC [184]. Unal et al. (2012) showed that AAW had the highest maternal serum concentration of bisphenol A (BPA), 10-fold higher than CAW, while Hispanic women had intermediate concentrations with an increasing trend to higher concentrations compared to Caucasian women, demonstrating significant racial/ethnic differences in maternal/fetal BPA concentrations [185]. Mandrup et al. (2016) indicated that low-dose exposure to BPA can affect mammary gland development in male and female rats, causing increased growth within ducts, which may be accompanied by an increased risk of developing hyperplasic lesions, similar to early signs of BC in women [186]. After exposure to BPA, the normal-like human breast epithelial cell line, MCF-10F, displayed an increased expression of BRCA1/2, BARD1, CtlP, RAD51 and BRCC3, which are all associated with DNA repair, in addition to the suppression of PDCD5 and BCL2L11 (BIM), which are involved in cell death [187].

Moreover, Black children experience much larger increases in BMI, weight and height compared to White children, while Mexican–American children are placed between Black and White children [188]. Persistent organic pollutants (POPs) bioaccumulate in adipose tissue, resulting in greater body burdens of these environmental toxicants with obesity [189]. For example, Black people experience higher exposure levels to polychlorinated biphenyls (PCBs) compared to White people [190]. A cross-sectional study found that AA subjects consumed more fish than white subjects [191]. PCBs, known for their high lipophilicity and persistence, tend to bioaccumulate in organisms through the food network, accruing in fish and marine mammals’ adipose tissue, where the PCB concentration is thousands of times higher than in water due to the biomagnification process [192]. Leng et al. (2016) showed that several PCBs are abundant in both human serum and breast tissue and would increase the risk of BC [164].

### 5.4. Migration Patterns and Breast Cancer Disparities

Migrants are influenced by different risk factors before, during and after migration [193]. Lamminmäki et al. (2023) reported that non-Western immigrant women in Nordic countries, Denmark, Finland, Iceland and Norway, had statistically significantly lower BC incidence than native women, but the BCR among immigrant women increased with the duration of residence [193]. Interestingly, these authors specified that higher education increased the BCR among immigrant women [193]. Similarly, BC rates of occurrence are four–seven times greater in America than in China and Japan. Once women from Asian countries like Japan, China, or the Philippines migrated to the United States, their BCR rates climbed over multiple generations, becoming practically similar to the BCR of U.S. White people [194]. Herbach et al. (2021) also identified disparities in BC progression related to nativity; immigrants from Asia, Eastern Europe, Latin America and the Caribbean and developing or transitional nations had higher disparities compared with immigrants from developed countries that experienced the least disparity [195].

Thus, immigrants’ BCR increased compared to people remaining in the countries of origin, primarily due to exposure to a Western lifestyle [194], especially to a Western non-healthy diet, as a risk factor for the development and preservation of the long-term inflammation of tissues correlated to innate immune cell reprogramming [196]. The inflammation linked to a lifestyle is a significant factor in the initiation, development and progression of BC [197]. The “metaflammation” concept reflects the crosstalk between the immune landscape, metabolic pathways, obesity and metabolic syndrome (MetS), resistance to insulin and persistent inflammation [198]. It is commonly established that MetS is a risk component for or indicator of BC and is more common in patients with BC [199,200,201].

## 6. Breast Cancer Disparities Related to Developmental Disorders

Cancer starts when the first cell undergoes a harmful mutation [202]. During sexual reproduction, fertilization is the union of two gametes, the oocyte and sperm, to form a diploid zygote and to initiate the development of a new and unique embryo [203]. The oocyte differentiates and starts to develop within a primordial follicle of the embryonic ovary of the future mother. Herein, the oocyte is a resting cell in which DNA damage accumulates over time until follicle recruitment and ovulation due to the absence of mechanisms to eliminate the failed cells during replication [204]. Apart from germline mutations in BC susceptibility genes, which could be inherited by the oocyte, it is also subject to the detrimental effects of the mother’s aging [205]. Inherited mutations are called germline mutations because they are present in gametes, both in the ovum and sperm, and become generally present in every cell of the resulting child’s body [206]. Germline mutations increase susceptibility to tumors, while somatic mutations are the secondary reason for the occurrence of cancers [206]. In addition, gametes represent targets for EDCs and thus a way for environmentally induced alterations/epimutations with transgenerational inheritance over several generations [207].

Interestingly, de novo genetic mutations accumulate even with the first zygotic cell divisions [208], so in-womb development represents a “sensitive window” for the introduction of mutations because of a higher rate of cellular proliferation [209]. A human cell must repair over 10,000 DNA lesions per day to counteract the intrinsic causes of DNA damage, but it is also necessary to consider the lesions induced by environmental sources of DNA damage [210]. Consequently, the failure to detect and repair such lesions at the cell level can lead to a harmful mutation rate, genomic instability or cell death [210]. Furthermore, the embryonic genetic mosaicism that arises early in development as a consequence of the mutational landscape is implicated in cancer [209]. As shown above, a small proportion of cancers are due to inherited mutations, which result in a high risk of developing specific cancers [211]. Mutations that occur in somatic cells are called somatic mutations, and they accumulate in healthy cells of the body throughout life, becoming an important cause of cancer that may change across a range of time periods, from one to fifty years, so the final neoplastic landscape of a malignant subclone within a tumor reflects the sum of acquired mutations in time by somatic evolution [212,213,214,215]. If we consider the mammary glands’ development, this process starts in the mother’s womb. Thus, there are three stages of mammary gland development in humans: embryonic, pubertal and adult [216]. The development of mammary glands starts in the embryonic ectoderm during embryogenesis, with the formation of milk/mammary lines that resolve into mammary placodes, which expand and invaginate within the underlying mesenchyme to form mammary buds, followed by the formation of the initial ductal tree present at birth [217]. Thus, during embryonic mammary development, normal breast cells proliferate, migrate and invade the stromal compartment, similar to BC cells that proliferate, suffer the EMT, invade and migrate from the primary tumor site to distant sites to form organotropic metastases [217].

Evidence suggests that estrogen levels are higher in AAW compared with CAW [218], so an embryo’s exposure to excessive maternal endogenous and/or synthetic estrogens, i.e., endocrine disruptor chemicals, could be associated with an increased risk of malignant transformation of the breast tissue later in life [219]. Soto et al. (2008) hypothesized that fetal exposure to xenoestrogens may play a role in the increased incidence of BC and suggested that BC may begin in the womb and impact the early stages of growth of the breast ducts [220,221]. In addition, Cohn et al. (2015) showed that in utero exposure to dichlorodiphenyltrichloroethane (DDT) is associated with an increased risk of BC, mainly in Africa and Asia where DDT exposure persists and use continues [222].

Phthalates, phenols and parabens are temporary EDCs linked to breast cancer [223]. Biomarker concentrations of temporary EDCs vary more among women versus men and among Black Americans than White Americans, owing to insufficient access to healthy food or the use of specific goods with greater amounts of phthalates, such as relaxers for hair and skin-whitening topic products, which are specifically marketed to Black consumers [223]. It has been demonstrated that AA are also predominantly exposed to excessive amounts of bisphenol A (BPA), as indicated by the fact that urine BPA levels across Black people of all ages were substantially higher than those in the non-Black population [224]. Furthermore, Tchen et al.’s (2022) findings support that exposure to BPA and bisphenol F (BPF) in pregnant women is associated with disruption of aromatic amino acid, xenobiotic, steroid and other amino acid metabolisms, which are connected to responses to stress, regulation of weight, steroid metabolism, inflammation and reproduction [224]. 

Wormsbaecher et al. (2020) linked the exposure of EDCs to molecular alterations that develop over time and contribute to an increased susceptibility to BC in adulthood by identifying significant dysregulated genes and transcriptional modifications in mature fibroblasts subjected to BPA in the uterus and diethylstilbestrol (DES), along with particular extracellular matrix (ECM) compositions and increased collagen deposition in adult mammary glands [225]. Evidence suggests that elevated breast density is a strong BC risk factor because collagen fiber features may be associated with BC risk and progression [226]. It is known that women with the highest breast density have an estimated four–five-fold greater risk of developing BC compared to women with the lowest breast density [227]. Many authors have shown that Black women have a statistically significantly higher absolute breast area density (40.1 cm^2^) compared with White women, who have 33.1 cm^2^ [228]. Moreover, black women also have a higher volumetric density (263.1 cm^3^) than White women (181.6 cm^3^) [228]. Caswell et al. (2013) showed that women with Ashkenazi Jewish ancestry are more likely to have age-adjusted and body mass index (BMI)-adjusted percent mammographic density (PMD) due to a unique set of genetic variants or environmental risk factors that increase mammographic density [229].

Exposure to BPA through inhalation or body accumulation can also regulate estrogenic signals, which in turn can cause cancer cells to proliferate and become malignant by activating the Wnt/β-catenin pathway. This pathway is widely linked to the pathophysiology of advanced BC and to the development of embryos [230].

After birth, pubertal development and reproductive life events, such as pregnancies, lactation and mammary involution accomplished by physiological apoptosis, have been described as subsequent stages of mammary gland development. Puberty initiates branching morphogenesis, which requires estrogen, growth hormone (GH), and insulin-like growth factor 1 (IGF1), to create a ductal tree in the fat pad, while during pregnancy, progesterone and prolactin generate alveoli development, leading to milk secretion during lactation. Also, dramatic changes that occur in the mammary gland during each pregnancy are orchestrated by signaling pathways that regulate a specialized subpopulation of mammary stem and progenitor cells [217]. Black women have more children, especially at younger ages, and a lower prevalence of breastfeeding than White women [179], which have been associated with a higher incidence of ER-/PR- BC in AA women relative to White women [231]. In the United States, Black teens overall have higher pregnancy and birth rates than White teens [232]. AA girls experience earlier menarche [233], which is also established as a risk factor for BC [234]. Moreover, Nguyen et al. (2019) found that parity and a young age at first pregnancy have been associated with a significant reduction in the risk of developing the luminal subtype of BC, but not TNBC [235].

## 7. Microbiomics- and Metagenomics-Related Disparities in Breast Cancer

The human microbiome is “the second genome of the body” [236], accounting for over 3.3 million genes [236], as well as our “last organ” [237]. Thus, the human microbiome includes all specific types of microbiota and extremely complex interactions between them, such as the bacteriome, archaeome, mycobiome and virome, studied by microbiomics [237]. Metagenomics studies microorganisms from specific environments by functional gene screening or sequencing analysis [238], being a collection of genomes and genes from microorganisms, resulting in bacterial, archaeal, fungal and viral metagenomes [237]. Due to its importance to human health, the human microbiome has become a focal point for precision medicine [239].

Human breast tissue and milk harbor unique and diverse microbiota, partially translocated from the gastrointestinal tract [240] as well as from the skin as another putative source of pathogenicity to breast tissue [241]. It is known that the microbiota inhabiting the breast tissue TME is involved in breast carcinogenesis [242]. Additionally, women with BC at the post-menopausal stage and healthy controls have different gut microbiota compositions and functions, which may have an impact on BC development [243]. Wang et al. (2017) showed that breast tissue revealed significantly different microbiomes in BC and non-BC patients, with a decreased abundance of *Methylobacterium* in cancer patients [240]. Moreover, BC patients harbor urinary microbiomes abundant in Gram-positive bacteria associated with skin flora [240]. Recently, Niccolai et al. (2023) documented the presence of a sexually dimorphic breast-associated microbiota, defined as a “microgenderome” [244]. These authors observed that, in women, the dysbiosis extends to the whole breast tissue, whereas in men, it appears to be present just in the tumor site [244].

Certain breast or gastrointestinal microorganisms, which are found in altered equilibrium/dysbiosis, may create toxins that harm DNA, break down the proteins released from tumor suppressor genes, cause oxidative stress (OS), activate pro-inflammatory mechanisms, and alter cell proliferation, survival pathways and the immune system [245]. Moreover, since estrogens are the most important risk factor in BC, especially in postmenopausal women, an important role of the human microbiome is the regulation of steroid-hormone metabolism [236]. Thus, the enzymes of intestinal microorganisms deconjugate conjugated estrogen metabolites, leading to a biologically active form of estrogen that arrives back in the bloodstream, or synthetize estrogen-like compounds that mimic estrogen function [236]. Other evidence suggests that bacteria can invade and transform BC cells, inducing cytoskeleton rearrangements and promoting metastatic colonization [246,247]. Thus, the EMT and inflammation are the molecular mechanisms that are most frequently affected by pathogenic organisms to induce malignant progression [248]. Conversely, the eubiosis state acts as a protective factor against cancer [245].

Smith et al. (2019) emphasized that the microbial communities in the breast tissue of non-Hispanic Black (NHB) and non-Hispanic White (NHW) women can differ by race, stage or BC subtype in [249]. A study conducted by Price et al. (2022) detected that the gut microbiome profiles differ between Black and White women in association with insulin sensitivity [250]. Thus, 50% of Black women have been classified as insulin-resistant compared to 30% of White women [250]; Black women also have a greater relative abundance of *Actinobacteria* compared with White women [250]. It is known that secondary metabolites derived from *Actinobacteria* have an influential role in tumor development as well as inhibition [251]. Usually, patients with breast cancer typically show alterations in the composition of microbes in their breasts in addition to decreased microbial diversity [252] of gut microbiota [245]. Moreover, studies have demonstrated significant differences in the relative abundance of specific taxa between NHB and NHW women [252]. Both tumor and normal tissue adjacent to tumor (NAT) samples emphasized a specific microbiota in both NHB and NHW women, whereas, when compared to a matching NAT area, the microbial diversity in NHB TNBC cancer tissue was much reduced [252]. Smith et al. (2022) reported that TNBC has a specific microbiota that differs from the less aggressive BC subtypes, emphasizing a correlation between host metabolic process changes and breast microbial dysbiosis in NHB and NHW women’s breast cancers [253]. Thus, potential race-specific microbial biomarkers of BC correlate to genes involved in tumor aggressiveness, angiogenesis, migration and metastasis, as well as oncogenic signaling pathways GLI and Notch in a specific manner [36].

## 8. Conclusions

Precision oncology is based on deep knowledge of the molecular profile of tumors and allows for more accurate and personalized therapy for specific groups of patients. Evidence suggests that different biomarkers have been found to have racial and ethnic differences, among other types of disparities, such as chronological or biological age-, sex/gender- or environmental exposure-related ones. Usually, BC disparities are due to ethnicity, socioeconomic position, psycho-social stressors, comorbidities, and a Western lifestyle. The aim of this review was to deepen the understanding of BC-related disparities, mainly from a biomedical perspective that includes genomic-based differences, disparities in breast tumor biology and developmental biology, differences in breast tumors’ immune and metabolic landscapes, ecological factors involved in these disparities, as well as microbiomics- and metagenomics-based disparities in BC.

Black women disproportionately bear the burdens of BC. Triple-negative breast cancer (TNBC) is twice as prevalent among Black women compared with White women. BC occurs in either gender, but female breast cancer (FBC) is the main cancer among women worldwide, while male breast cancer (MBC) is a rare disease. However, male patients have worse survival and higher mortality compared to FBC patients. Older women have a worse prognosis compared to younger patients. In Black women, a younger age and obesity, associated with a low socioeconomic status, influence TNBC development. Breast tissue revealed significantly different microbiomes in BC and non-BC patients, while a sexually dimorphic breast-associated microbiota, defined as a “microgenderome”, is involved in male–female BC disparities. Moreover, multiple studies have demonstrated that many chemicals have disproportionately high exposure levels to Black women and emphasize BC-associated biological activities, leading to higher exposure-related biomarker levels compared to White women. As for eastern immigrant women in Nordic countries, Denmark, Finland, Iceland and Norway following their migration to the U.S., women from Asian nations, such as China, Japan and the Philippines, saw an increase in their BCR rates over several generations, eventually matching those of U.S. White women. 

Thus, *BRCA 1/2* germline pathogenic variants are highly ethnic-specific in BC patients, with a high frequency of *BRCA* variation in specific countries or ethnic groups, especially within genetically isolated populations, as well as other somatic and germline mutations in high- or moderate-prevalence genes, such as *TP53*, *PI3KCA*, *CHEK2*, *BARD1*, *ATM* and *PALB2*, which also emphasize racial differences. Moreover, the frequency of the presence of hormone receptors within BC varies by race and ethnicity. Also, TME, insulin growth factor (IGF) signaling, tumor necrosis factor (TNF)-mediated pathways, xenobiotic metabolism, metabolic reprogramming, epigenetic mechanisms/hypermethylation, obesity and metaflammation all contribute to high race/ethnic disparities.

Androgen-receptor (*AR*) gene copy increase, steroid hormone-mediated pathways, especially estrogen and AR-related pathways, DNA methylation levels of cytokeratin-related genes and other genes involved in cytoskeleton architecture, membrane trafficking, gene transcription, cell migration, invasion, adhesion, survival and growth, and sexually dimorphic breast-associated microbiota (known as the “microgenderiome”) contribute to biological sex/gender-related disparities in BC.

Genomic instability, steroid hormone-mediated pathways, transcriptome changes, telomere shortening, epigenetic alterations, deregulating nutrient sensing, mitochondrial dysfunction, loss of proteostasis, altered intercellular communications, chronic inflammation, Wnt/β-catenin signaling and composition and functions of gut/breast microbiota contribute to age-related disparities in BC.

We can conclude that onco-breastomics, in principle, based on genomics, proteomics, epigenomics, hormonomics, metabolomics and exposomics data, is able to characterize the multiple biological processes and molecular pathways involved in BC disparities, clarifying the differences in incidence, mortality and treatment response for different groups of BC patients.
